# Editorial: Nanocellulose: A Multipurpose Advanced Functional Material

**DOI:** 10.3389/fbioe.2021.738779

**Published:** 2021-07-22

**Authors:** Muhammad Wajid Ullah, Orlando J. Rojas, Ronan R. McCarthy, Guang Yang

**Affiliations:** ^1^ Department of Biomedical Engineering, Huazhong University of Science and Technology, Wuhan, China; ^2^ Bioproducts Institute, Departments of Chemical and Biological Engineering, Chemistry and Wood Science, The University of British Columbia, Vancouver, BC, Canada; ^3^ Division of Biosciences, Department of Life Sciences, College of Health and Life Sciences, Brunel University London, Uxbridge, United Kingdom

**Keywords:** nanocellulose, synthesis, characterization, functionalization, tissue engineering, additive manufacturing, textile, environment

Nanocellulose refers to the cellulosic materials having at least one dimension at the nanoscale. It is the most abundant natural polymer on Earth, extracted from plants termed plant cellulose (Yadav et al., 2021), produced by microbial cells called bacterial cellulose (BC) or bacterial nanocellulose (BNC) (Ul-Islam et al., 2021), and synthesized enzymatically such as by the cell-free enzyme systems, named as bio-cellulose (Ullah et al., 2015; Kim et al., 2019). Over the past few decades, the different forms of nanocellulose, including cellulose nanocrystals (CNCs), cellulose nanofibers (CNF), and BNC have received tremendous attention for the development of innovative materials owing due to their abundance, renewability, and remarkable structural and physical properties such as high surface area and special surface chemistry, high crystallinity and mechanical strength, hydrophilicity, moldability, polyfunctionality, and excellent biological features (biocompatibility, biodegradability, and non-toxicity). These properties could be tuned through the addition of other natural and synthetic polymers, nanomaterials, clays, and other materials, as well as through the incorporation of additional functional groups such as peptides (Malheiros et al., 2018). Unlike CNCs and CNF, the structural features of BC could be tuned by varying the growth and culture conditions of the cellulose-producing microbial cells (Ullah et al., 2016). The surface chemistry, porosity, fiber orientation, and physical structure of nanocellulose can be controlled at macro-, micro-, and even nanoscales. Different types of nanocellulose in the form of gels, sheets, films, membrane, pellets, filaments, fibers, papers, tubes, capsules, sponges, laminates, and coatings find novel and emerging applications in food (Cazón and Vázquez, 2021; Haghighi et al., 2021), tissue engineering (Du et al., 2019), wound dressing (Mao et al., 2021; Wang et al., 2021), drug delivery (Li et al., 2018; Raghav et al., 2021), bioinks for 3D printing (McCarthy et al., 2019; Fourmann et al., 2021), biosensors (Farooq et al., 2020), energy storage devices (Sheng et al., 2019), membrane filters (Yuan et al., 2020), textiles (Salah, 2013), flexible displays (Fernandes et al., 2009), facial masks (Bianchet et al., 2020), and several others.

The global environmental degradation issue, the depletion of natural energy resources, the health-related problems, and other human needs are greatly pushing the material-related research towards the use of various polymeric materials from renewable resources like plants (i.e., cellulose, hemicellulose, lignin) and microorganisms (i.e., BNC) for fabrication of functional materials for different applications. Although nanocellulose obtained from such sources possesses unique features, it does not possess features like antimicrobial activity, antioxidant activity, electromagnetic properties, and catalytic activity, which are required for its specialized applications. Furthermore, nanocellulose also possesses limited biocompatibility and optical transparency. The plant cellulose, although a cheap source, requires complex extraction procedures and post-synthesis processing as it contains hemicellulose, lignin, and some minerals. In contrast, the BC, although pure, is expensive and produced at a low yield by the microbial cells. Altogether, all types of nanocellulose require complicated functionalization procedures for tuning their features to meet the demands of desired applications. To date, various studies have been conducted for cost-effective synthesis, processing, and structural modifications of nanocellulose; there is still much to be explored about its facile and low-cost synthesis, tuning its properties and establish structure-function relationship, and explore its innovative and emerging applications.

Taking the advantages of unique surface chemistry and physical structure of nanocellulose, this Research Topic in Frontiers in Bioengineering and Biotechnology, Sections “Biomaterials” and “Nanobiotechnology” entitled “Nanocellulose: A Multipurpose Advanced Functional Material” is aimed to discuss the pilot-scale production, unveil the structure-function relationship, and identify the innovative and emerging applications of different types of nanocellulose. This Research Topic features a total of 20 articles, including fourteen original research articles, a brief research report, four reviews, and one systematic review, contributed by the experts in the field. These articles are mainly focused on the synthesis, characterization, and applications of various forms of nanocellulose in different fields like biomedical (antibacterial, wound dressing, bone tissue engineering, drug delivery, nerve tissue engineering), pharmaceutics, additive manufacturing, food, environment, textile, and optoelectronics.

It has been recognized that the ‘production cost’ and ‘prices of BC-based products’ are two indispensable aspects hindering the commercialization of nanocellulose. To this end, the review by Zhong from Hainan Yeguo Foods Co. Ltd., Hainan, China, rationally demonstrated the feasibility of industrial-scale production by elaborating the industrial setup and discussing the production cost of BC. The article took a case-by-case approach and discussed the market value of various BC-based products for their use in the food, personal care, biomedical, and textile industries. Wang et al. also took on industrial-scale production of nanocellulose, with a focus on producing cellulose nanowhiskers (CNWs), having similar morphology to CNCs, through acid hydrolysis by using low-cost equipment made of common stainless steel, where the used acid was effectively recovered at the end of the process. These CNWs could serve as the effective rheology modifier for their use in the preparation of inks for 3D printing. The emerging 3D printing technology, in contrast to the widely used fabrication techniques of developing nanocellulose-based composites, is providing new avenues based on ‘polymer printing’ for obtaining biological scaffolds. A systematic review by Tarassoli et al. identified the potential of different biomaterials used in extrusion printing and identified that alginate, poly (ε-caprolactone) (PCL), gelatin, and methacrylated gelatin are the most commonly used natural polymer. They realized a shift from synthetic to natural bioinks for biological applications, although the qualitative analysis did not show any link between the type of bioink, the extrusion printing technique, and the target tissue, thus indicating the infancy of this fabrication approach. Although at an early stage of development, the potential merits of 3D printing technology are quite clear, and further requires the formulation of tissue-specific bioinks with balanced printing materials, viable cells, growth factors, and finally the selection of an appropriate bioprinting technique for printing of tissue constructs for targeted applications, which would ultimately lead to maturation of 3D printing technology. As the future of printing technology mainly relies on the selection of unique and novel biomaterials, this systematic overview of different natural and synthetic polymers will provide solid grounds in the pursuit of choosing a suitable polymeric system for targeted applications. Moreover, the clogging caused by the cellulose fibers during the extrusion printing needs to be resolved while keeping in view that the fibrous morphology of biological scaffolds is advantageous for adhesion and proliferation of cells and have a similarity with the biological tissues such as collagen.

As indicated in the title of this Research Topic, nanocellulose is a multipurpose functional material that finds applications in different areas; a large portion of papers in this Research Topic covers different application areas. As nanocellulose serves as a matrix for a range of materials, Wang et al., Khattak et al., and Revin et al. took on developing nanocellulose-based composites with different antimicrobial materials, including gold nanoclusters, silver sulfadiazine, and sodium fusidate by developing films, hydrogels, and aerogels, respectively. These nanocomposites showed antibacterial activity against different clinically relevant microorganisms, such as *Escherichia coli*, *Streptococcus mutans*, *Staphylococcus aureus*, and *Pseudomonas aeruginosa*, which are generally responsible for pathogen burdens at the wound site. Thus such nanocellulose-based antimicrobial nanocomposites could be useful for the development of wound dressing materials. Continuing with the broad-spectrum biomedical applications of BC owing to its non-toxic nature and ability to allow the reinforcement of a variety of materials, Sajjad et al. also took on developing BC-based antimicrobial wound dressings for treating partial thickness skin burns by using a natural product, curcumin, as the reinforcement material having antimicrobial, antioxidant, antineoplastic, and wound healing capabilities and a long history of medical use. In addition to these, several other materials of different sizes, shapes, physiology (liquid and solid), and origin, having antimicrobial activities, have been successfully impregnated into cellulose matrix with the aim to develop antimicrobial materials for biomedical applications. A review by Zheng et al. summarized several wound dressings of nanocellulose with organic antimicrobials (natural polymers, bioactive materials, synthetic materials), inorganic nanomaterials (metal/metal oxides, carbon-based nanomaterials, nanosilicates), and antibiotics. All these studies indicate the usefulness of different types of nanocellulose to serve as a matrix for the impregnation of antimicrobials, thus could be used as a platform for the local and controlled delivery of therapeutics against multidrug-resistant bacteria, a major threat to health care systems globally due to extensive overuse of antibiotics.

Despite the high crystallinity and mechanical strength of nanocellulose, it does not possess the desired strength of a natural bone; nevertheless, this limitation can be effectively overcome by making its composite with other materials, thanks to its unique matrix, to induce the formation of calcium phosphate for bone tissue engineering. Hong et al. reinforced the surface-oxidized CNCs with PCL to induce the formation of calcium phosphate. The 3D printed CNC/PCL nanocomposite demonstrated enhanced mechanical strength, crystallinity, and thermal stability. Furthermore, the scaffold supported the growth of MC3T3 preosteoblasts, thus indicating its biocompatible nature. Altogether, the 3D CNC/PCL scaffolds could be a suitable candidate for bone tissue engineering applications. The mechanical, thermal, and biological features of nanocellulose could also be tuned by making its composites with different materials. A perspective review by Khan et al. discussed that, in addition to serving as a matrix for doping of minerals and bioceramics for developing bone and cartilage substitutes, nanocellulose could also be used as a drug carrier for treating bone-related diseases. The review explicitly described the structure-function relationship of nanocellulose with bone tissues. While there is no doubt about the biocompatible and non-toxic nature of nanocellulose, there is still much to be investigated about its *in-vivo* degradation and unveiling the associated complications. Moreover, nanocellulose as a drug carrier has some limitations due to its hydrophilic nature and only serves as a carrier for hydrophilic drugs especially when used in the form of a hydrogel, following the solubility principle ‘like-dissolve-like’. Badshah et al. developed a nanocellulose-based drug delivery system for oral administration. Considering the insolubility of nanocellulose in common organic solvents, BC was dissolved in ionic solvent *N*-methyl-morpholine-oxide and loaded with model drugs, famotidine or tizanidine, which was abruptly released. Although this system could be useful for oral administration of the drug, a sustained and prolonged drug release is desired in some situations. A controlled drug release from nanocellulose could be achieved through its chemical modification, such as with pH-sensitive and electrically conductive materials, which would allow a sustained drug release under the influence of external pH and electrical stimuli, respectively. Nerve tissue engineering is another important application area of nanocellulose-based biomaterials. The development of nanocellulose-based biomaterials capable of stimulating neural tissue repair is in demand owing to the limited regenerative capacity of neural tissues, with neurons as the most noticeable. Towards this goal, Xu et al. developed a spongy electrical conduit of hydroxyethyl cellulose with soy protein and polyaniline and evaluated its regeneration potential of peripheral nerve for treating sciatic nerve injury in a rat model. In addition to the direct use of nanocellulose in tissue engineering or as a carrier for drugs, bioactive materials, and nanomaterials, the nanocellulose-based scaffolds are also used as useful tools in the diagnosis of cancer models (Ul-Islam et al., 2019). Kim et al. developed a bioink comprised of gastric tissue-derived decellularized extracellular matrix (g-dECM) with cellulose nanoparticles and used it as a 3D cell printing-based gastric cancer model. The high mechanical strength of the cellulose nanoparticles imparted stability to the 3D printed scaffold, which in turn better supported the progression of gastric cancer.

The use of nanocellulose in optoelectronics is another exciting area of research. Considering the renewable nature of nanocellulose, it is highly demanding to study its optical behavior and conductive properties to explore its applications in this area. For instance, the portable and wearable electronics requiring highly thermo-conductive but electrically insulating film, the development of nanocellulose-based films with conductive materials could be a good choice considering the innate non-conductive nature of nanocellulose. In contrast to the conventional sustainable approach of combining boron nitrate sheets with regenerated cellulose, which usually produces brittle structures of low toughness, Xu et al. developed a dual crosslinked and anisotropic regenerated cellulose and boron nitrate nanosheets, where the partial chemical bonding interactions enabled the interfiber slippage and prevented the mechanical fracture while the non-covalent hydrogen bonding interactions served as the sacrifice bonds and dissipated the stress energy, and thus led to the development of high mechanical strength and tough nanocellulose-based sheets. Santos et al. also focused on the optoelectronics applications of nanocellulose by developing freestanding CNCs film with silica through the chiral nematic organization. Considering the transparent or semitransparent nature of different forms of nanocellulose, the optical properties of films were tuned by varying the ratio of CNCs and silica. The incorporation of light-emitting Rhodamine 6G allowed the complimentary control of the optical properties of the films. These freestanding luminescent and iridescent CNCs-based films could be useful materials for applications in the development of optical devices like lasers, filters, and sensors. The optical transparency of cellulose can be enhanced in various ways such as acetylation (Gonçalves et al., 2016) and making its composite with transparent materials like poly (2-hydroxyethyl methacrylate) (Di et al., 2017). Likewise, Kim et al. enhanced the optical transparency of CNCs films through reinforcement of polyurethane. The change in optical transparency was determined by measuring the change in the refractive index, which was mainly associated with the alignment of CNCs by dielectrophoresis. Unlike the above-mentioned studies of developing transparent nanocellulose films for wound dressing applications, the developed transparent CNCs films by tuning the refractive index in the presence of polling electric field could be used as a tunable optical lens. Moreover, a study by Tao et al. showed that the self-assembly of CNCs in a tilted cuvette demonstrated simultaneous reflection of left-handed and right-handed circularly polarized light and formed rainbow color CNCs-based films. This phenomenon could be employed in the synthetic development of one-dimensional chiral photonic materials with patterned and ambidextrous reflection for photonic applications.

Nanocellulose also finds useful applications in the textile industry and environment sector. It serves as a matrix for immobilization of catalysts, enzymes, and other sensing materials not only to sense environmental pollutants but also degrade different wastes, for example, wastes from the textile industry, which could be further used as the carbon source and biotransformed into value-added products (Zhang et al., 2021). A review by Felgueiras et al. discussed the various technologies in practice or in the phase of development for the production of nanocellulose-based textiles. Huang et al. synthesized biocompatible fluorescent carbon dots from cellulose, which detected mercury at a small detection level and with high specificity. Ul-Islam et al. developed BC-based multipurpose green nanomaterials with *Aloe vera* hydrogels for biomedical (adhesion and proliferation of osteoblast cells) and environment (adsorption of metal ions) applications.

In conclusion, this Research Topic not only discusses the fundamental knowledge of nanocellulose synthesis, its unique properties, and multipurpose applications in different fields but also provides the base for the production of nanocellulose-based innovative materials for novel and emerging applications and discusses the pilot-scale production and commercialization of nanocellulose-based products.
